# Tribological Properties of Polyamide 46/Graphene Nanocomposites

**DOI:** 10.3390/polym14061139

**Published:** 2022-03-12

**Authors:** Pyoung-Chan Lee, Su Young Kim, Youn Ki Ko, Jin Uk Ha, Sun Kyoung Jeoung, Donghyeok Shin, Jung Hoon Kim, Myeong-Gi Kim

**Affiliations:** 1 Materials Technology R&D Division, Korea Automotive Technology Institute, Cheonan-si 31214, Korea; pclee@katech.re.kr (P.-C.L.); sykim1@katech.re.kr (S.Y.K.); ykko@katech.re.kr (Y.K.K.); juha@katech.re.kr (J.U.H.); skjeoung@katech.re.kr (S.K.J.); 2 R&D Center, Woo-Sung Chemical Co., Ltd., Cheonan-si 31214, Korea; sdh@metapoly.co.kr; 3 R&D Center, BESTGRAPHENE Co., Ltd., Yeoju-si 12616, Korea; un8827@best-graphene.com

**Keywords:** wear, polyamide 46, graphene, self-adsorption

## Abstract

Polyamide 46 (PA46) is used in various automotive parts because of its excellent heat resistance and mechanical properties. This study aims to improve the frictional properties of PA46 using the lubricating ability of graphene. Nanocomposites are prepared via two mixing methods: Graphene powder is compounded directly with PA46 pellets through a twin-screw extruder, or PA46 powder is added to graphene dispersion for self-adsorption, and subsequently, it is dried and compounded with PA46 through the twin-screw extruder. Application of the nanocomposite in the friction field is evaluated via the pin-on-disk method. The coefficient of friction of the nanocomposite prepared by self-adsorption is lower than that of the nanocomposite prepared by direct compounding. The mechanical properties of the nanocomposite fabricated by self-adsorption are superior to those of other materials. This can be attributed to the uniform dispersion of graphene and the strong attractive force between the PA46 matrix and graphene.

## 1. Introduction

Polyamide (PA) is used in the automotive industry as a representative engineering plastic. Polyamide 46 (PA46) exhibits higher crystallinity, melting temperature, and thermal stability than the commonly used polyamide 6 (PA6) and polyamide 66 (PA66) because of its high amide content per unit length chain length and its symmetrical chain structure. Further, PA46 exhibits excellent wear properties because of its high crystallinity, which incentivizes its application in highly heat-resistant frictional parts, such as valves and gears [1,2,3]. Recently, PA46 composites have been used to improve wear resistance caused by the high performance and non-lubrication of automotive parts. Studies on the mechanical and frictional properties of PA46-based fiber-reinforced composites are being conducted. Gordon [3] studied the wear properties of aramid fiber-reinforced composites, whereas Kurokawa [4] investigated the wear properties of carbon fiber-reinforced composites.

Graphene is a two-dimensional single layer of graphite, and it is an emerging two-dimensional carbon-based nanomaterial. Graphene exhibits excellent thermal properties, electrical conductivity, mechanical strength, moisture barrier properties, and wear resistance. Consequently, graphene-polymer nanocomposites are expected to have numerous potential applications [5,6,7,8,9]. Studies on the variation in the mechanical and frictional properties of the composites of graphene and various polymers, such as polyacrylonitrile [9], PA6 [10], and polyetherether ketone [11] based on the content of graphene are being actively conducted. The dispersibility of graphene in the polymer matrix needs to be ensured to realize the excellent physical properties of graphene-reinforced nanocomposites.

In this study, graphene was composited in various ways to improve the wear resistance of PA46. A chemically modified graphene (CMG) dispersion was used to increase the dispersibility of graphene, and composite particles were prepared and processed using PA46 nanoparticles and by exploiting self-adsorption behavior. For comparison, a composite was prepared by directly compounding graphene powder with PA46 pellets. The frictional properties were compared and analyzed based on the manufacturing method.

## 2. Materials and Methods

### 2.1. Materials

Graphene powders of BGF-H grade (non-oxidized graphene, lateral size of 2 µm or less, thickness of 3 nm or less) and BGF-R grade (reduced graphene, lateral size of 5–7 µm, thickness of 20–30 nm) were provided by BESTGRAPHENE (Yeoju-so, Korea). For the production of functionalized graphene, 150 μm grade graphite flakes purchased from Graphene Supermarket were used. Stanyl^®^ TW341 grade from DSM (Heerlen, Netherlands) was used to produce PA46.

### 2.2. Preparation of Chemically Modified Graphene (CMG)

Chemically modified graphene (CMG) was designed to substitute -OH, -COOH, and epoxide groups of graphene oxide with -CN, amide, and amine through functionalization to enable molecular structural interaction with PA46. An improved, recently developed, and widely used method was used to produce graphene oxide (GO) [12]. At 50 °C, 150 μm grade graphite flakes (30 g, 1 wt. equiv.) were added to a mixture of H_2_SO_4_/H_3_PO_4_ (3600 mL:400 mL) and KMNO_4_ (180 g, 3 wt. equiv.) at a 9:1 ratio and stirred for 12 h. The reaction mixture was cooled to room temperature (23 °C) and diluted by immersing it in ice water (4000 mL) containing 30% H_2_O_2_ (3 mL) after the reaction was completed. The non-oxidized graphite was removed by filtration, whereafter the oxidized graphite was precipitated by centrifugation at 4000 rpm for 2 h. The precipitate was washed a minimum of two times in a mixed solution of DI water (2000 mL), 30% HCl (2000 mL), and ethanol (2000 mL), and subsequently, it was freeze-dried to obtain fine non-agglomerated particles. Distilled water (2000 mL) and oxidized graphite (2 g) were mixed and sonicated for a minimum of 4 h using an ultrasonic grinder to obtain an aqueous solution of GO.

A graphene surface-modification reaction was performed using 4,4-oxidianiline to obtain a chemically modified graphene colloid that can undergo physicochemical interactions with PA46. A 500 mL aqueous solution of 0.1% GO was prepared by sonicating for a minimum of 2 h using an ultrasonic homogenizer. The aforementioned solution was mixed with a solution of 20 g of 4,4-oxidianiline in 500 mL of N,N-dimethylformamide (DMF). This mixture was stirred at 90 °C for 40 h. Thereafter, the mixture was cooled to room temperature (23 °C), a precipitate was separated by centrifugation, washed three to five times with acetone, and filtered to obtain graphene. The as-obtained graphene was immediately immersed in ethanol, and the positive charge (-N^+^) of the substituted 4,4-oxidianiline generated an electrostatic repulsive force between the graphene monolayers to yield a chemically modified graphene colloid with high dispersibility.

### 2.3. Preparation of Nanocomposites

Nanocomposites were fabricated using two methods. In the first method, PA46 pellets were injected into the main feeder of a twin-screw extruder (L40/D19, BAUTEK, Pocheon-si, Korea), graphene powder was injected into the side feeder, and the two feeds were compounded at 300–330 °C. Samples with graphene powder (BGF-H, BGF-R) contents of 0.05, 0.1, and 0.2 wt% (sample names DH, DR, respectively) were prepared. In the second method, finely ground (D_50_ = 385 μm) PA46 powder was mixed with a graphene dispersion, precipitated through chemical self-adsorption, and dried (24 h, 100 °C oven) to prepare a master batch (MB, graphene content was 0.5 wt%). The MB was injected into the main feeder of the twin-screw extruder with PA46 pellets. The graphene content was adjusted to 0.05, 0.10, and 0.15 wt% by adjusting the ratio of 0.5 wt% MB and PA46 pellets (sample name S05). The mixing ratios are listed in Table 1.

### 2.4. Characterization

Functionalized graphene was analyzed via Raman spectrometry (Confotec MR350, SOL instruments, Minsk, Belarus), transmission electron microscopy (HR-TEM, JEM-3010, JEOL, Tokyo, Japan), zeta potentials measurement (LITESIZER 500, Anton Paar, Graz, Austria), and FT-IR (Fourier Transform-Infrared Spectroscopy, Spectrum Two, PerkinElmer, Waltham, MA, USA). A pin-on-disc tester (THT, Anton Paar, Graz, Austria) was used to determine the frictional properties of the material. The testing was conducted at 25 °C, a motor speed of 400 RPM, a test load of 10 N, and by using a stainless-steel ball of 6 mm diameter as the pin. Following the frictional evaluation, the frictional surface was analyzed using an optical microscope (VHX-700F, Keyence, Osaka, Japan). The tensile strength, flexural strength, and flexural modulus were measured using a universal testing machine (UT-100F, MTDI Korea, Daejeon, Korea).

## 3. Results

### 3.1. CMG Analysis

**Figure 1:**
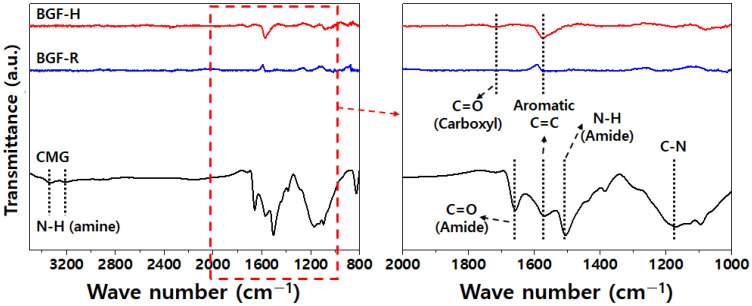
FT-IR spectra of graphene powder (BGF-H, BGF-R) and CMG.

Figure 1 shows the FT-IR spectra of the graphene powder (BGF-H, BGF-R) and the CMG. BGF-H (non-oxidized graphene) and BGF-R (reduced graphene) exhibit a few functional groups that can be observed using FT-IR spectroscopy. For CMG, the FT-IR spectra exhibit absorption peaks that correspond to the amide and amine groups. Bands with peak values at around 1650 and 1437 cm^−1^ are assigned to the amide vibrations. Peaks between 3330 and 3300 cm^−1^ were attributed to the secondary amide group. The C-N stretching vibration gave rise to bands in the region 1200–1100 cm^−1^.

**Figure 2:**
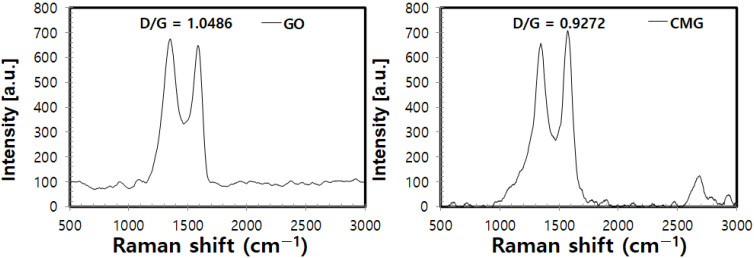
Raman spectra of GO and CMG fabricated by the improved method.

The structural characteristics of the as-synthesized graphene oxide and CMG were identified through their Raman spectra. In the Raman spectrum of graphene, a D peak is caused by defects within the crystal and the edge of the sample, whereas a G peak is attributed to the vibration of the hexagonal crystal structure composed of sp2 carbon atoms commonly seen in graphite-based materials. Figure 2 shows the Raman spectra of GO and CMG produced by the improved method wherein the characteristic D band (near 1360 cm^−1^) and G band (near 1580 cm^−1^) peaks of graphene can be identified [13]. The 2D band (near 2700 cm^−1^) was identified in the spectrum of CMG that underwent reduction and surface-functionalization. The D/G ratio decreased to 0.927 for CMG compared to 1.049 for GO. The smaller D/G ratio of CMG than that of GO indicates that reduction and functionalization decrease defects in the CMG structure, which increases crystallinity. This suggests the formation of a crystalline structure that possesses the functionality of graphene. Since the 2D peak near 2700 cm^−1^ is caused by the double resonance of a phonon with the energy of 1350 cm^−1^, the particular peak appears around 2700 cm^−1^, which is double the energy of 1350 cm^−1^. In the case of multilayered graphene, various scattering processes take place because of the higher number of energy bands, and this increases the number of 2D peaks and thereby leads to a broad 2D band [14]. As shown in Figure 2, no 2D peak was observed in GO because the average size of the sp2 domain was small in the presence of defects. A clear 2D peak appeared in CMG because of the increased average size of the sp2 domain and crystallinity through reduction and functionalization.

**Figure 3:**
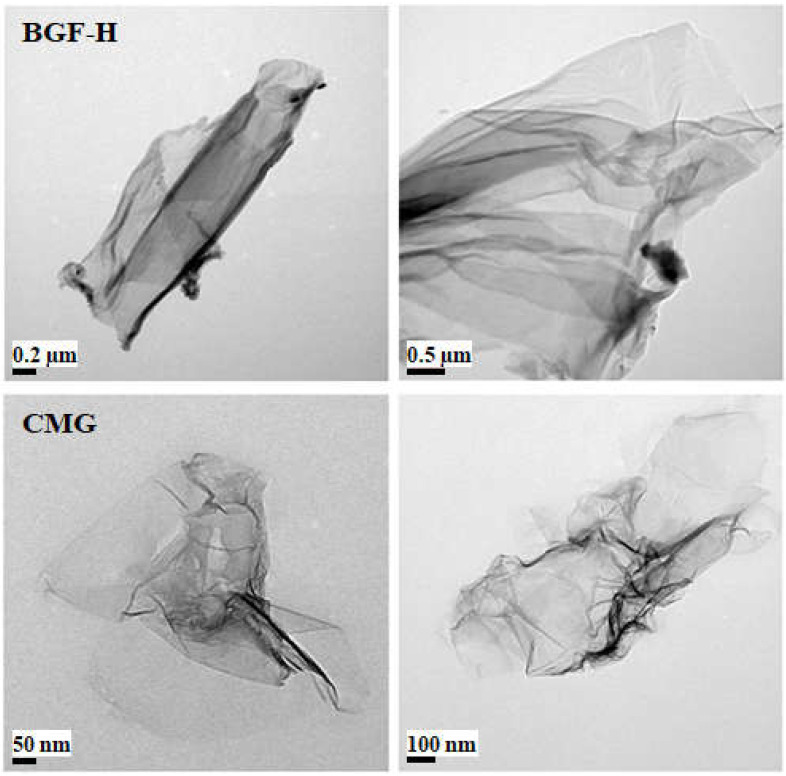
TEM observation of non-oxidized graphene (BGF-H) and CMG.

**Figure 4:**
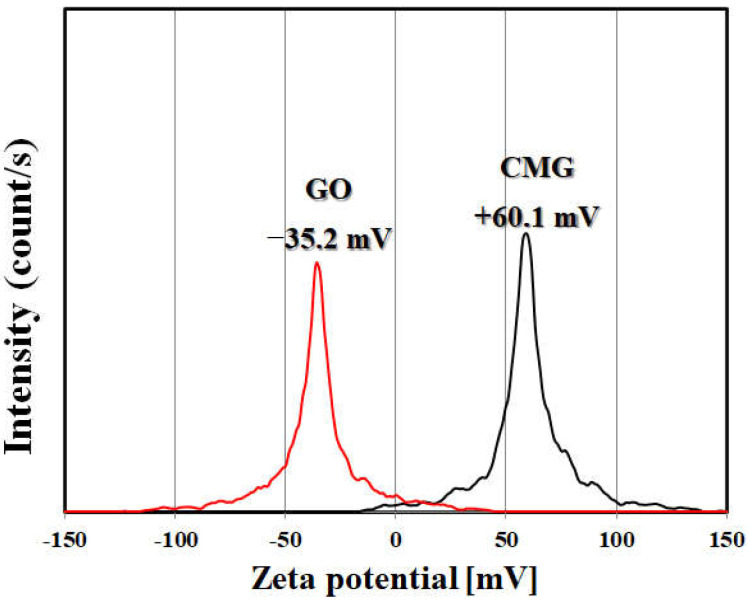
Zeta potential measurements of GO and CMG fabricated by the improved method.

Figure 3 shows a TEM image of non-oxidized graphene BGF-H and CMG. Non-oxidized high-quality graphene with a single-to-multiple-layer structure is difficult to manufacture. However, CMG has a strong positive charge because 4,4-oxidianiline is substituted in the molecular structure of graphene through the reduction and functionalization of graphene oxide. The electrostatic repulsion between the particles physically separates them. The stability of the dispersion is maintained by forming a high-quality structure with single or multiple layers. Figure 4 plots the zeta potential measurement of GO and CMG for the zeta potential of GO produced by the improved method −35.2 mV, whereas that of CMG produced by the reduction and functionalization was +60.1 mV. The intensity of the zeta potential is determined according to the intensity of the surface charge of the particle. Therefore, CMG has a very high positive charge. The dispersibility is high at the zeta potentials of ±30 mV or higher. Therefore, the CMG synthesized in this study has very high dispersion stability, which results in a single-to-multiple-layered graphene structure.

**Figure 5:**
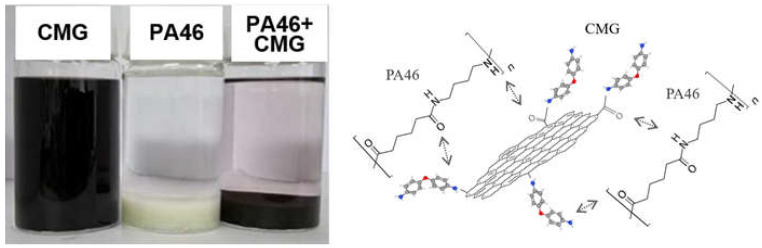
Photographs and schematic representation of the self-adhesion behavior of graphene on PA46 powder.

Figure 5 shows the photographs of CMG dispersion, PA46 powder in ethanol, and PA46 nanoparticles mixed in CMG dispersion. The amide and amine groups on the graphene surface were self-adsorbed and precipitated on the PA46 surface by hydrogen bonding with the amide group of PA46 when PA46 powder was added to the evenly dispersed CMG dispersion.

### 3.2. Frictional Behavior

The number of layers of graphene is the main parameter in basic research conducted to improve frictional properties using graphene. Interactions between graphene layers occur through van der Waals forces. The mechanical properties of graphene change significantly as the number of graphene layers increases because van der Waals forces have low bonding strength compared to the covalent bonds connecting carbon atoms in individual planes [12]. Previous studies have suggested that the number of graphene layers has a dramatic effect on the frictional force. The frictional force increases with an increase in the number of graphene layers. However, the rate of increase differs significantly between different studies. In the presence of strong bonding between graphene and the base material, the frictional force has been reported to decrease with an increase in the number of layers [15,16].

**Figure 6:**
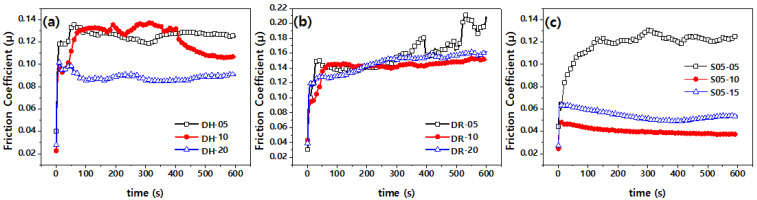
Friction coefficients of composites with different contents of graphene: (**a**) GH, (**b**) GR, and (**c**) S05.

Figure 6 plots the change in the friction coefficient with the graphene content and method of mixing. The plot for polymer nanocomposites illustrates that there are two major trends in frictional behavior. In the initial stage of friction, the friction coefficient significantly increases because of the strong friction between the steel ball and the specimen. Under friction, the friction coefficient gradually decreases and stabilizes with the surfaces of the two parts in contact, which become smoother. This behavior is already known for composites: When the load and wear are moderate, lubricant materials do not reach the surface in sufficient quantity, and this insignificantly alters the friction and wear [17]. 

The effect of the friction coefficient can be examined from Figure 6 based on the difference in lateral size and thickness. In terms of lateral size, BGF-R is the largest, and BGF-H and CMG are of equal size. The thickness of the BGF-R is the largest, followed by those of BGF-H and CMG. The specific surface area of CMG is the largest and followed by those of BGF-H and BFG-R. Graphene lubricates because of its molecular structure. Therefore, the higher the specific surface area, the lower the friction coefficient is expected to be. Consequently, at a particular lateral size, the lower the number of layers, the lower is the friction coefficient. In addition, for a smaller number of layers, the amount of graphene is greater at a particular content, and this increases the probability of the expression of the characteristics of graphene. Figure 6a,b shows that the frictional force increased as the number of layers increased, which agrees with the results of previous studies. That is, BGF-R, which has a larger number of layers (Figure 6b) than BGF-H, has a larger friction coefficient (Figure 6a). 

The friction coefficient of the S05 sample compounded through the self-adsorbing powder was smaller than that of the GH and GR conditions directly compounded with the polymer matrix, as shown in Figure 6c. The CMG appears to have a strong attractive force toward PA46, a polymer matrix, and it reduces the frictional force.

**Figure 7:**
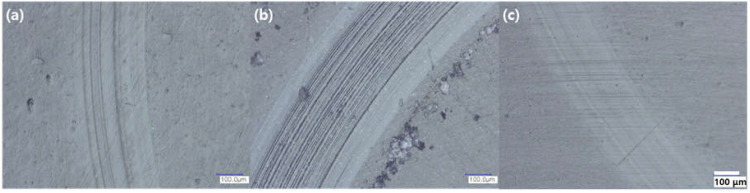
Optical micrographs of worn surfaces of (**a**) DH-10, (**b**) DR-10, and (**c**) S05-10 under friction condition (load: 10 N, sliding speed: 400 RPM, duration: 10 min).

Figure 7 shows an optical microscope image of the frictional surface of the 1.0 wt% graphene content sample in the dry state. The wear properties of other nanocomposites exhibited a similar trend to those of the samples in Figure 7. Friction wear is a combination of abrasive and adhesive wear. Polishing and smoothing are the major types of damage sustained by polymer surfaces during friction, and this occurs when soft and hard surfaces rub against each other. In the wear mechanism, the polymer is moved to a harder mating surface by the close contact of two materials, and thereafter, they are removed as abrasive debris [18,19]. As shown in Figure 7b, the DR-10 sample has an uneven wear surface with large abrasive grains. This undergoes strong friction with the substrate after large-sized abrasive particles are peeled off and form grooves of different depths on the substrate surface, which exhibits low wear resistance and indicates that abrasive and adhesive wear are the principal wear modes [10]. Figure 7a is similar to Figure 7b, although no abrasive grains were observed in the former, which can explain why the DH sample exhibited higher wear resistance than that of the DR sample. In contrast, as shown in Figure 7c, the surface of the nanocomposite manufactured through self-adsorption exhibited some signs of wear and no abrasive particles. The defect could not have extended to the periphery because CMG was firmly bonded to the surface owing to the protective and lubricating effect on the matrix. This ensures higher wear resistance [10]. The lubricating properties are increased when the termini of graphene and the polymer matrix form a physical and chemical bond. Defects may occur when there is insufficient bonding force, and this can lead to the deterioration of the frictional properties.

### 3.3. Mechanical Properties

**Figure 8:**
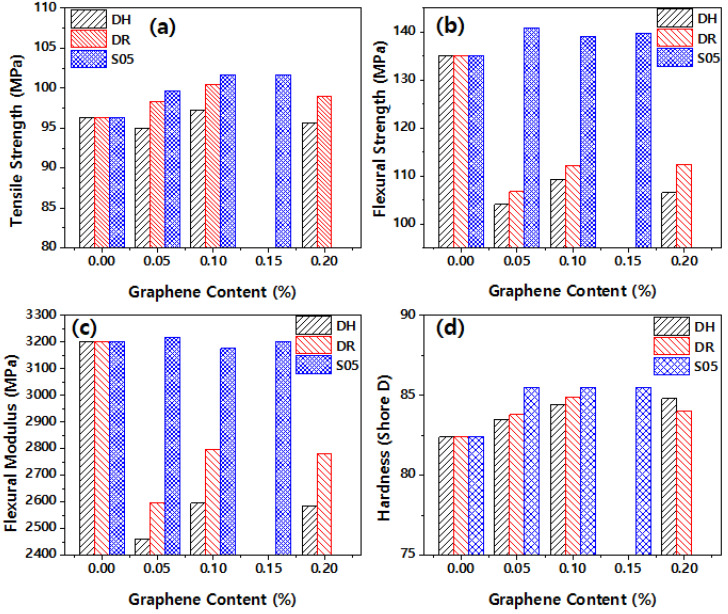
Mechanical properties of the different content of graphene: (**a**) Tensile strength, (**b**) flexural strength, (**c**) flexural modulus, and (**d**) hardness.

Figure 8 plots the mechanical properties according to the mixing method and graphene content. As shown in Figure 8a, the material blended with powdered functional graphene after self-adsorption exhibited higher tensile strength than the material directly blended with graphene powder. This difference can originate from the uniform dispersion and strong bonding between the matrix and graphene [10]. Figure 8b,c plots the flexural strength and flexural modulus, respectively. As shown in Figure 8b,c, flexural properties are greatly reduced in the samples (DH, DR) directly compounded with graphene powder. This decrease may have occurred because of the low attractive force between graphene and the polymer chain [18,19]. However, in the sample (S05) containing CMG prepared by self-adsorption, the flexural strength and flexural modulus were increased. This may have occurred because of the strong attraction between graphene and the polymer matrix PA46.

Figure 8d shows that the Shore D hardness increased with the increase of graphene content. The hardness value of S05-15 reaches the maximum value of 85.5, which is 3.6% higher than pure PA46. This increase in the hardness of S05 sample is attributed to the increased rigidity of the molecular chain by the strong attraction between the matrix and graphene [10]. The improved hardness would reduce the initial deformation of the corresponding specimen, and it would thus affect the actual contact area during friction [20,21]. Owing to the hardness of graphene and strong attraction between the matrix and CMG, PA46/graphene nanocomposites, S05-10, and S05-15, exhibited improved wear resistance.

The uniform dispersion of the nanoadditives and the attractive force between graphene and the matrix are critical to the mechanical properties of nanocomposites. Figure 8 shows that the method of preparing a nanocomposite by compounding after passing through the primary adsorption process with PA46 powder in the graphene dispersion state resulted in superior mechanical properties compared to the method of preparing a nanocomposite by compounding graphene powder directly with PA46 pellet. Graphene powder is difficult to delaminate by melting because of the strong attractive force between graphene layers during processing, and it reduces the efficacy of the additive. The powdered graphene composite may have facilitated the dispersion during melting after being adsorbed on the surface of PA46 powder.

Figure 8 shows that mechanical properties differ with the lateral size and thickness of the graphene sheets. The attractive force between the surface of the additive and the polymer matrix is critical to the mechanical properties of nanocomposites. The lateral size of the H grade is smaller than that of the R grade. The thickness is lower, and the surface area is larger, which facilitates the creation of numerous interfaces with the matrix polymer. However, the mechanical properties appear to be lower than those of the R grade because of the lower attractive force between interfaces.

## 4. Conclusions

The frictional and mechanical properties of graphene-reinforced polyamide 46 were investigated. The uniform dispersion of the nanoadditives and attractive force between the graphene and matrix are critical to the frictional and mechanical properties of nanocomposites. The dispersibility and compatibility with the matrix were higher when the graphene was self-adsorbed onto the PA46 surface. Thereafter, it was subjected to secondary melt processing when the graphene powder was directly compounded. Thus, the friction coefficient decreased by more than 50%. The findings of this study may guide the application of PA46 composite materials in friction-driving parts to reduce the weight of automotives.

## Figures and Tables

**Table 1:** Formulation of PA46/Graphene Nanocomposites.

Sample	PA46	BGF-H	BGF-R	MB	Graphene Content (%)
REF	100	-	-	-	-
S05-05	90	-	-	10	0.05
S05-10	80	-	-	20	0.10
S05-15	70	-	-	30	0.15
DH-05	99.95	0.05	-	-	0.05
DH-10	99.90	0.10	-	-	0.10
DH-20	99.80	0.20	-	-	0.20
DR-05	99.95	-	0.05	-	0.05
DR-10	99.90	-	0.10	-	0.10
DR-20	99.80	-	0.20	-	0.20

## Data Availability

The data presented in this study are available on request from the corresponding author.
